# ACE2-independent infection of T lymphocytes by SARS-CoV-2

**DOI:** 10.1038/s41392-022-00919-x

**Published:** 2022-03-11

**Authors:** Xu-Rui Shen, Rong Geng, Qian Li, Ying Chen, Shu-Fen Li, Qi Wang, Juan Min, Yong Yang, Bei Li, Ren-Di Jiang, Xi Wang, Xiao-Shuang Zheng, Yan Zhu, Jing-Kun Jia, Xing-Lou Yang, Mei-Qin Liu, Qian-Chun Gong, Yu-Lan Zhang, Zhen-Qiong Guan, Hui-Ling Li, Zhen-Hua Zheng, Zheng-Li Shi, Hui-Lan Zhang, Ke Peng, Peng Zhou

**Affiliations:** 1 CAS Key Laboratory of Special Pathogens & State Key Laboratory of Virology, Wuhan Institute of Virology, Chinese Academy of Sciences, Wuhan, People’s Republic of China; 2 University of Chinese Academy of Sciences, Beijing, People’s Republic of China; 3 State Key Laboratory of Genetic Engineering, School of Life Sciences, Zhongshan Hospital, Fudan University, 200438 Shanghai, China; 4 Center for Organoid and Regenerative Medicine, Greater Bay Area Institute of Precision Medicine (Guangzhou), 511462 Guangzhou, China; 5 Department of Respiratory and Critical Care Medicine, Tongji Hospital, Tongji Medical College, Huazhong University of Science and Technology, Jie Fang Road, Han Kou District, 430030 Wuhan, China

**Keywords:** Immunology, Microbiology

## Abstract

SARS-CoV-2 induced marked lymphopenia in severe patients with COVID-19. However, whether lymphocytes are targets of viral infection is yet to be determined, although SARS-CoV-2 RNA or antigen has been identified in T cells from patients. Here, we confirmed that SARS-CoV-2 viral antigen could be detected in patient peripheral blood cells (PBCs) or postmortem lung T cells, and the infectious virus could also be detected from viral antigen-positive PBCs. We next prove that SARS-CoV-2 infects T lymphocytes, preferably activated CD4 + T cells in vitro. Upon infection, viral RNA, subgenomic RNA, viral protein or viral particle can be detected in the T cells. Furthermore, we show that the infection is spike-ACE2/TMPRSS2-independent through using ACE2 knockdown or receptor blocking experiments. Next, we demonstrate that viral antigen-positive T cells from patient undergone pronounced apoptosis. In vitro infection of T cells induced cell death that is likely in mitochondria ROS-HIF-1a-dependent pathways. Finally, we demonstrated that LFA-1, the protein exclusively expresses in multiple leukocytes, is more likely the entry molecule that mediated SARS-CoV-2 infection in T cells, compared to a list of other known receptors. Collectively, this work confirmed a SARS-CoV-2 infection of T cells, in a spike-ACE2-independent manner, which shed novel insights into the underlying mechanisms of SARS-CoV-2-induced lymphopenia in COVID-19 patients.

## Introduction

Since its emergence in December 2019, SARS-CoV-2, the etiology of coronavirus disease 2019 (COVID-19), quickly spread to the majority of countries in the world and posed great threats to public health. The virus shares 79.5% genome identity with SARS-CoV-1 and also uses angiotensin-converting enzyme 2 (ACE2) as a cell entry receptor.^1–5^ Typical clinical symptoms of COVID-19 patients include fever, fatigue, dry cough, and pneumonia, whereas around 20% of the severe cases may die of multi-organ failure.^6–9^

Apart from the respiratory system, multiple organs including the immune system of COVID-19 patients were also targeted by SARS-CoV-2 infection. Notably, lymphopenia was observed in 83.2% of the patients on admission, and fatal infections were associated with more severe lymphopenia over time.^6–8^ Lymphocytes (particularly T cells) play a central role in the human immune system, a decrease of which would result in immune suppression and serious complications.^10^ It has been proposed that viral-induced lymphopenia might be due to direct infection, cytokine-mediated cell death, tissue sequestration of lymphocytes, or suppression of the bone marrow or thymus for T-cell generation.^11^ In the case of MERS-CoV, apoptosis induced by direct viral infection of T cells has been observed in vitro, which possibly explained lymphopenia in MERS patients.^11^ SARS-CoV-1 viral particles were also observed in multiple leukocytes from an autopsy study, suggesting that direct infection might account for the decrease in lymphocytes.^12^ Similarly, SARS-CoV-2 particles or proteins were also found in the spleen and lymph nodes from a study of 91 deceased COVID-19 cases, suggesting an infection of lymphocytes.^13^ Furthermore, in COVID-19 immune landscape depicted by single-cell RNA-seq studies, SARS-CoV-2 viral RNA has been found in multiple immune cells, including myeloid cells with phagocytic activity (neutrophil and macrophage) and lymphocytes without phagocytic activity (T, B, and NK cells).^14,15^ Notably, SARS-CoV-2 RNA-positive immune cells did not co-express the entry factors ACE2 and TMPRSS2, or other hypothesized entry co-factors.^14,15^ It is speculated that cell-associated SARS-CoV-2 viral positivity may represent a mixture of replicating virus, immune cell engulfment, and virions or virally infected cells attached to the cell surface.^14,15^

It has been shown that SARS-CoV-2-infected human monocytes, monocyte-derived macrophages, and dendritic cells in vitro, which potentially plays a major role in COVID-19 pathogenesis.^16,17^ However, whether SARS-CoV-2 infects lymphocytes, which do not express ACE2, to result in lymphopenia is still unknown. This knowledge gap also brings difficulty for our understanding of how lymphocytes lost the ability to control viral infection. Here, we provided evidence that activated T lymphocytes could be infected by SARS-CoV-2 in an ACE2-independent manner. The infection leads to pronounced T-cell apoptosis in vitro or in patients with COVID-19. Our findings shed light on the understanding of SARS-CoV-2 infection-induced lymphopenia.

## Results

### Presence of SARS-CoV-2 in lymphocytes from patients with COVID-19

**Fig. 1:**
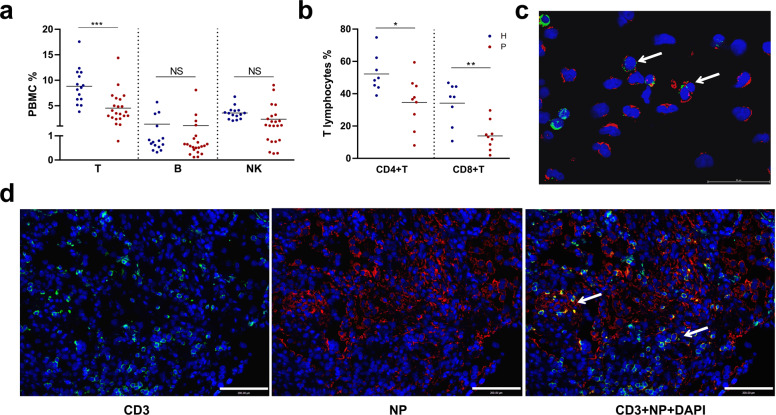
Peripheral blood lymphocytes are infected by SARS-CoV-2 in COVID-19 patients. **a** Percentage of different types of lymphocytes in the healthy donors (*n* = 15) or in COVID-19 patients (*n* = 22). (**b**) Percentage of CD4 + and CD8 + T lymphocytes in healthy donor (*n* = 8) or in COVID-19 patients (*n* = 9). **c**, **d** Immunofluorescent test of the presence of SARS-CoV-2 viral antigen in T cells. PBCs (**c**) or postmortem lung section (**d**) from COVID-19 patients were stained with T lymphocytes (CD3, green), SARS-CoV-2 (NP, red) and nuclei (DAPI, blue). In-house-made pAb against SARS-CoV-2 NP was used. White arrows indicate areas T lymphocytes that were infected by SARS-CoV-2. Pictures were taken under confocal microscopy with a bar = 50 μm (**c**) or 200 μm (**d**). Comparison of mean values (**a**, **b**) between two groups was analyzed by Student’s *t* test. *P < 0.05; **P < 0.01; ****P < 0.0001; NS no significance

Multiple immune cell types, including lymphocytes, have been shown enriched for SARS-CoV-2 viral RNA in multiple single-cell RNA-seq studies.^14,15^ To determine whether SARS-CoV-2 infects lymphocytes, we analyzed peripheral blood cells (PBCs) collected from COVID-19 patients. PBCs were prepared from 22 patients, who were all at severe condition during the study along with 15 healthy donors. We first analyzed major lymphocyte cell types including T (CD4 + helper T and CD8 + cytotoxic T), B, and natural killer (NK) cells for their population changes or the presence of viral antigen upon infection. For all patients tested, the ratios of blood T lymphocytes declined significantly compared to those in healthy donors, whereas B and NK cells appeared to be unaffected (Fig. 1a). Notably, CD4 + and CD8 + T lymphocytes almost declined to zero in some patients (Fig. 1b). The results suggested that lymphopenia in these patients is likely attributed to a decline of T lymphocytes.

We then analyzed the presence of SARS-CoV-2 viral antigens in PBCs using flow cytometry or by immunofluorescence assay (IFA). The results suggested that T lymphocytes were infected and in certain patient CD4 + T cells showed a high infection rate (Supplementary Fig. S1a). We also confirmed the presence of viral antigen in T lymphocytes from patient blood by immunofluorescence analysis (IFA) (Fig. 1c). Furthermore, we prepared postmortem lung sections from patients with a fatal infection and analyzed T lymphocytes infiltration and virus infection. We found T lymphocytes infiltration in the lung section, and many T lymphocytes were also positive for SARS-CoV-2 NP staining, indicating virus infection (Fig. 1d). A similar finding has also been reported.^13^ Taken together, we showed the presence of SARS-CoV-2 viral antigen in T lymphocytes either in the blood or in the lung section from the COVID-19 patients.

To further corroborate these findings, virus isolation was attempted from viral NP-positive PBCs. Patient PBCs were collected, determined for viral antigen using flow cytometry, and then co-cultured with Caco2 cells after three washes. Positive detection of viral RNA in the supernatant or viral protein in the Caco2 cells after co-culture indicated successful isolation and amplification of SARS-CoV-2 from PBCs of some COVID-19 patients (3 out of 5) but not from the healthy control (Supplementary Fig. S1b–e). Notably, in the three viral isolation positive samples, two also showed viral positive in the flow cytometry assay (P2 and P4), while the third one (P5) likely carried infectious virus at a level that was under the detection limit of flow cytometric analysis. Above all, we observed SAR-CoV-2 viral RNA and viral protein, and likely infectious virus in T lymphocytes from COVID-19 patients.

### SARS-CoV-2 infection of T cells in vitro

**Fig. 2:**
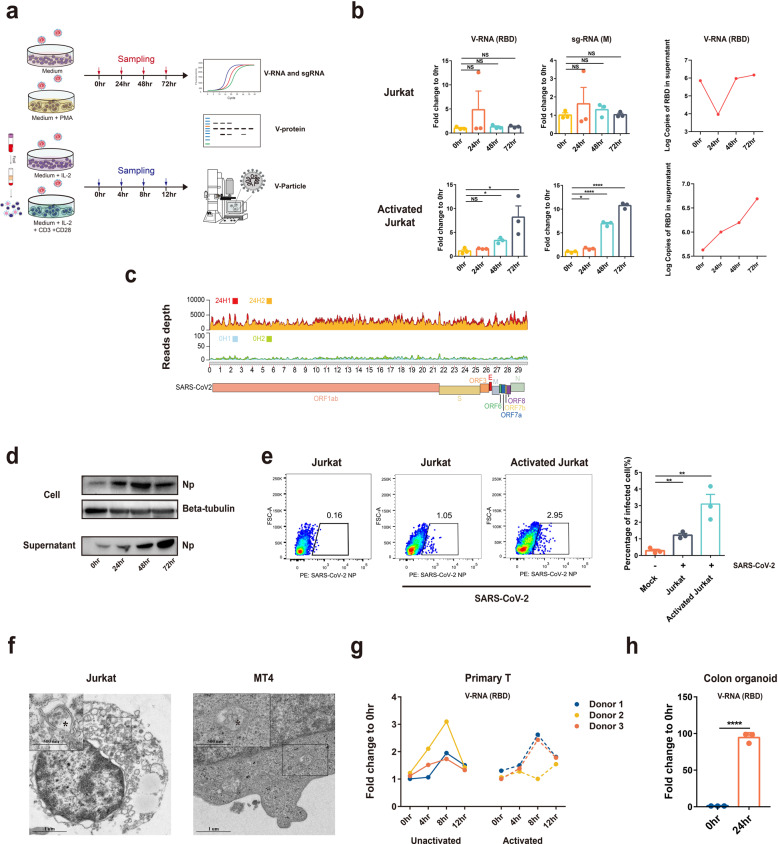
SARS-CoV-2 in vitro infection of T cell lines or primary T cells. **a** Schedule of experiments. **b** Unactivated or activated Jurkat cells were infected with SARS-CoV-2 (MOI = 0.1) and samples were collected at 0, 24, 48, and 72 h post infection. Viral load in cells or cell supernatant was then quantified by qPCR detection of total viral RNA (RBD of spike gene as target) or subgenomic RNA (sgRNA, M gene as target). **c** Depth and coverage comparison for SARS-CoV-2 0 h or 24 h-infected activated Jurkat cells. For each sample, virus quantity was normalized with its total reads number of sequencing. Two replicates are shown for each time point. **d** Viral NP in the infected activated Jurkat cells and cell supernatant was analyzed by western blot at 0, 24, 48, and 72 h post infection. **e** Viral NP 72 h-infected cells from (**d**) were analyzed by flow cytometry and the number of replicates that are represented in the bar graph is three. **f** Viral particles in infected activated Jurkat or MT4 cells were observed by transmission electron microscope. bar = 1 μm or 500 nm, magnification: 3500-folds and 9600-folds for Jurkat cell, 5000-folds or 11500-folds for MT4 cell. **g** Unactivated or activated primary T cells were infected with SARS-CoV-2 (MOI = 0.01) and samples were collected at 0, 4, 8, and 12 h post infection. Viral load in cells was then quantified by qPCR. **h** Colon organoids were infected with SARS-CoV-2 (MOI = 0.01). Zero hour and 24 h samples were harvested and quantified by qPCR. The data were analyzed by Student’s *t* test and statistical significance is indicated by the asterisks (*P < 0.05; **P < 0.01; ****P < 0.0001; NS no significance)

Since T lymphocytes population decreased in COVID-19 patients and CD4 + T lymphocytes showed a high viral antigen-positive rate, we then investigated whether SARS-CoV-2 infects CD4 + T lymphocytes. For this purpose, we conducted a serial of experiments to test whether SARS-CoV-2 infects T cells. Upon infection, both viral RNA detection targeting at the receptor-binding domain (RBD) and viral subgenomic mRNA (sgRNA) targeting at M gene were tested. Viral sgRNA is transcribed only in infected cells during viral replication and is not packaged into virions, and therefore indicates the presence of actively infected cells in samples. Viral nucleocapsid protein (NP) and viral particles were also detected using western blot (WB), flow or electron microscopy (EM). Jurkat or MT4 cells, two commonly used CD4 + T cell lines, and primary T cells isolated from healthy donors were infected with SARS-CoV-2 (Fig. 2a). In some experiments, T cells were also activated by Phorbol myristate acetate (PMA) for 2 h for Jurkat cells or by a combination of IL2 + CD3 + CD28 for 3 days for primary T cells before infection, considering a large proportion of T cells is activated in human (Supplementary Fig. S2).

At 0, 24, 48, and 72 h post infection, it was observed that SARS-CoV-2-infected Jurkat T-cell line in a time-dependent manner, and the infection was more robust in activated T cells. Accumulation of viral RNA and sgRNA in cells or viral RNA in the culture supernatant was observed (Fig. 2b). Next, we sought to determine whether the qPCR detection assay represents only partial viral genome replication. We performed RNA-seq analysis of the SARS-CoV-2-infected activated Jurkat T cells at 0 or 24 h p.i. and analyzed the viral reads depth and coverage across the viral genome. Compared to 0 h infected, a much higher depth of viral genomes (as high as 5000 reads depth) can be observed in the 24 h-infected cells, demonstrating an effective replication (Fig. 2c). We then determined viral antigens by WB and flow assay. Our results showed a time-dependent increased level of viral NP in cells or in the supernatant, similar to the findings in viral RNA detection (Fig. 2d, e). We further employed electron microscopy to analyze SARS-CoV-2 infection of T-cell lines. Activated Jurkat or MT4 cells were infected with SARS-CoV-2 for 72 h and viral particles with typical coronavirus morphology were observed in the cytoplasm of the infected cells (Fig. 2f). Finally, to corroborate the findings from T-cell lines, we tested the infectivity of primary T cells isolated from healthy donors. In the three donors, SARS-CoV-2 showed time-dependent infection of T cells that is peaked at 8 h, probably because of extensive cell death induced by the virus at this time point (discussed below). Activation sensitized the cells to SARS-CoV-2 infection in two of the three donors. As comparison, primary colon organoid was also infected, which showed much higher infection efficiency compared to T cells (Fig. 2g, h). Taken together, our data clearly show that SARS-CoV-2 could infect T cells in vitro, although at a lower efficiency compared to tissue cells.

### SARS-CoV-2 infection of T cells is ACE2 and TMPRSS2-independent

**Fig. 3:**
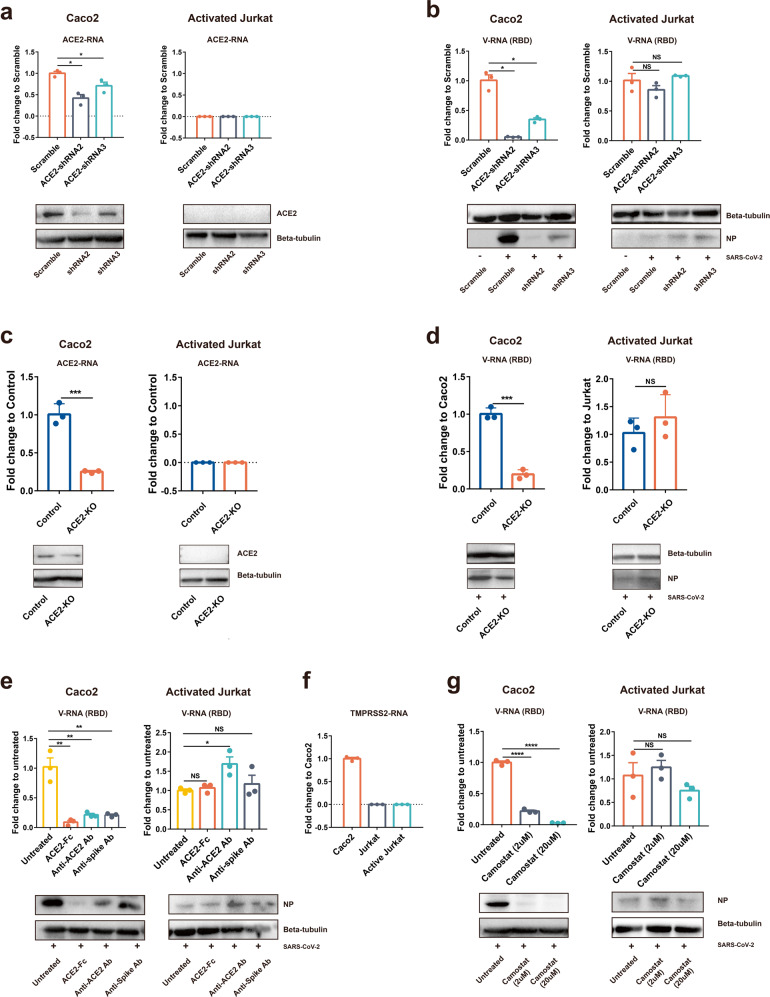
SARS-CoV-2 infection of T cell is spike-ACE2/TMPRSS2-independent. **a** The ACE2 expression level of Scramble or ACE2-knockdown Caco2 or Jurkat cells was analyzed by qPCR or WB. **b** ACE2 stably knockdown Caco2 or activated Jurkat cells were infected with SARS-CoV-2 (MOI = 0.01). Viral RNA or viral NP in cells was analyzed by qPCR or WB at 24 h post infection. **c** The ACE2 expression level of control or ACE-knockout Caco2 or Jurkat cells were quantified by qPCR or detected by WB. **d** ACE2 stably knock-out Caco2 or activated Jurkat cells were infected with SARS-CoV-2 (MOI = 0.01). Viral RNA or viral NP was detected using qPCR or WB. **e** For ACE2 blocking, Caco2 or activated Jurkat cells were pre-incubated with anti-ACE2 Ab (3.33 ng/μl final) before infected with SARS-CoV-2. For virus blocking, ACE2-Fc protein (10 μg/μl final) or RD#4-anti-Spike Ab (160 ng/μl final) were incubated with SARS-CoV-2 at a volume of 1:1 at 37 °C for 30 min. Cells were infected at 0.01 MOI for 24 h before they were quantified for SARS-CoV-2 viral RNA or NP protein by qPCR or WB. **f** The TMPRSS2 expression level of Caco2, Jurkat and activated Jurkat cells was analyzed using qPCR. **g** Caco2 or activated Jurkat cells were pre-incubated with Camostat (2 μM or 20 μM) for 1 h and then infected with SARS-CoV-2 (MOI = 0.01). Viral RNA or NP proteins were quantified. The results were derived from three independent experiments. Statistical analyses were carried out using Student’s *t* test (*P < 0.05; **P < 0.01; ****P < 0.0001; NS no significance)

It is generally believed that ACE2 is the entry receptor for SARS-CoV-2. However, major cell populations in PBCs express extremely low levels of ACE2, raising the question whether ACE2 also mediates SARS-CoV-2 virus entry of T cells. We first tested whether an ACE2 knockdown could dampen SARS-CoV-2 infection of T cells. The data showed ACE2 was successfully knocked down by ACE2-shRNAs in Caco2 cells. Jurkat T cells do not express detectable ACE2 under either mock or knocked down conditions (Fig. 3a). Correspondingly, ACE2 knockdown resulted in dramatically decreased SARS-CoV-2 infection in Caco2 cells but not in Jurkat T cells (Fig. 3b). To further confirm this finding, we did ACE2 knocked out in Caco2 and Jurkat cells (Fig. 3c). Similar to ACE2-knockdown cells, viral load decreased in Caco2-ACE2-KO cells but not in Jurkat-ACE2-KO cells (Fig. 3d). These results suggested that SARS-CoV-2-infected T cells in an ACE2-independent manner.

It was reported that soluble human ACE2 protein could block SARS-CoV-2 infection through competing virus binding with the cellular receptor.^3^ Thus, ACE2 antibody pre-incubated cells or spike antibody pre-incubated SARS-CoV-2 should also block viral infection, if the infection depends on spike-ACE2 binding. To analyze whether these molecules affect SARS-CoV-2 infection of T cells, we incubated virus with soluble human ACE2 protein or a commercial mAb targeting at RBD-ACE2 binding, or incubated cells with ACE2 blocking antibody before the infection of Caco2 or activated Jurkat T cells. The intracellular viral RNA was analyzed after infection. In Caco2, the three blockers strongly blocked SARS-CoV-2 infection, and ACE2 protein appears to be more potent than the other two treatments. In contrast, none of the three treatments affected the SARS-CoV-2 infection of Jurkat T cells (Fig. 3e).

Lastly, it is known that SARS-CoV-2 uses the serine protease TMPRSS2 for S protein priming before binding to ACE2 receptor, and a TMPRSS2 inhibitor has been approved for clinical use (Camostat) to block SARS-CoV-2 entry.^1^ The RNA expression of TMPRSS2 in Caco2, Jurkat, and activated Jurkat cells was determined by qPCR. The result suggested that neither unactivated nor activated Jurkat cell-expressed TMPRSS2 (Fig. 3f). We observed that Camostat inhibited SARS-CoV-2 infection of Caco2 cells in a dose-dependent manner. At a dose of 20 μm, Camostat almost completely blocked viral infection of Caco2 cells. In contrast, Camostat showed no inhibitory effect on SARS-CoV-2 infection of Jurkat T cells even at a high dose (Fig. 3g). Collectively, these results suggested that SARS-CoV-2 infection of T cells does not rely on the spike-ACE2/TMPRSS2 interaction.

### SARS-CoV-2 infection triggered T-cell death

**Fig. 4:**
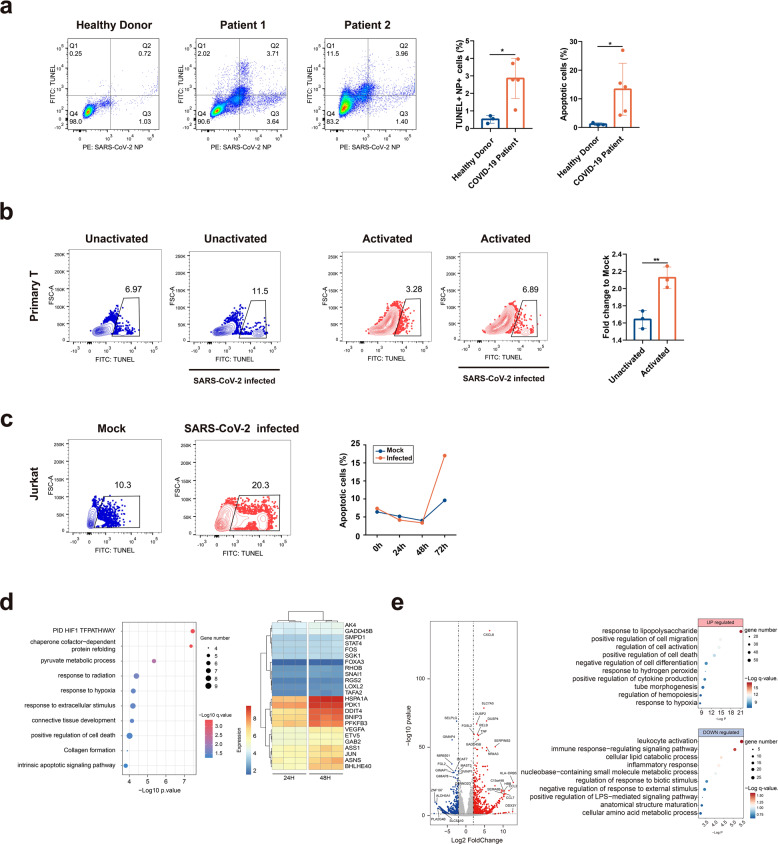
SARS-CoV-2 infection-induced apoptosis in T cells. **a** Detection of apoptotic T lymphocytes in human PBCs. PBCs were prepared from COVID-19 patients or from healthy donors. Apoptosis in virally infected T lymphocytes were determined using CD3, SARS-CoV-2 NP antibodies, and TUNEL assay. Detection results for two patients and one healthy donor, or the statistics of apoptotic cells or TUNEL/NP double-positive cells were shown for healthy donors (H, *n* = 3) or patients (P, *n* = 5). Comparison of mean values between two groups was analyzed by Student’s *t* test. *P < 0.05. **b** Unactivated or activated primary T cells were infected with SARS-CoV-2 (MOI = 0.01) for 8 h and cell apoptosis was analyzed with TUNEL assay. **P < 0.01. **c** Activated Jurkat cells were infected with SARS-CoV-2 (MOI = 0.01) for 0, 24, 48, and 72 h. The ratio of apoptotic cells in 72 h mock or SARS-CoV-2-infected cells were shown (flow chart). The ratio of apoptotic cells in different time points were also compared (plot). **d** Samples from C were subjected for RNA-seq analysis. The top ten upregulated GO pathways in 48 h compared to 24 h group are shown. Heatmap shows the normalized expression of genes that were enriched from PID HIF1 pathway. The data were analyzed by Student’s *t* test (*P < 0.05; **P < 0.01; NS no significance). **e** Compared to healthy donors, differentially **e**xpressed genes of severe COVID-19 patients were shown in the volcano plot, and the top ten differential expressed pathways were shown in the right panel

It is known that severe patients with COVID-19 showed marked decreased lymphocyte populations. To determine whether SARS-CoV-2 infection contributes to T-cell death, we tested PBC T lymphocytes apoptosis collected from patients with COVID-19. T lymphocytes from patients or from healthy donors were dual-labeled with a CD3 antibody and a viral NP antibody, and apoptosis was analyzed with the TUNEL assay. T lymphocytes from COVID-19 patients underwent pronounced apoptosis compared to those from the healthy donors, showing a more than tenfold increase of apoptotic cells. In some patients, most of the apoptotic cells were also viral antigen-positive (e.g., 65% in patient 1), suggesting viral infection played a role in peripheral blood T lymphocytes death in these patients (Fig. 4a).

To confirm the role of viral infection in T-cell death, we experimentally infected primary T cells isolated from healthy donors. With or without activation, cells were experimentally infected with SARS-CoV-2 for 8 h and apoptosis was analyzed with TUNEL assay. It can be observed that SARS-CoV-2 infection induced pronounced apoptosis in infected T cells compared with the mock-treated cells. Activation sensitized T cell to viral infection, as shown by higher apoptotic cells in the activated group (Fig. 4b).

Finally, we determined the cellular responses in T cells upon SARS-CoV-2 infection by bulk RNA-seq analysis. Activated Jurkat T cells were infected with SARS-CoV-2 for 0, 24, 48, and 72 h before they were collected for TUNEL assay. It can be observed that virus induced significant apoptosis at 72 h post infection, compared to mock-infected or cells at other time points (Fig. 4c). We then determined the dynamic cellular responses in cells that have been infected for 24 or 48 h, as the cells in 72 h groups contained too many dead cells and were not suitable for RNA-seq analysis. Compared to the 24 h group, the hypoxia-related GO pathways are significantly upregulated in 48 h group, including “PID HIF1 TF pathway”, “response to hypoxia”, “positive regulation of cell death”, and “intrinsic apoptotic signaling pathway”. It has been shown that SARS-CoV-2 infection triggers mitochondrial ROS production, which induces stabilization of hypoxia-inducible factor-1a (HIF-1a) in monocytes.^16^ Similarly in T cells, multiple genes involved in this oxidative stress response were upregulated: BNIP3, PFKFB3, FOS, JUN, BHLHE40, GADD45B, PDK1, and DDIT4 (Fig. 4d). To corroborate the findings in T cell lines, we conducted RNA-seq analysis to primary peripheral blood mononuclear cells (PBMCs) collected from three healthy donors and three severe COVID-19 patients. Our data showed an upregulation of cell responses to stimuli, cell death, or response to hypoxia pathways, and a down-regulation of leukocytes activation and signaling pathways, similar to the findings in the T-cell line (Fig. 4e). In summary, SARS-CoV-2 infection induced pronounced T-cell death, which is probably dependent on mitochondria ROS-hypoxia pathways.

### Exploration of potential receptors in T cells

Since our results suggested that the infection of SARS-CoV-2 to Jurkat T cell is ACE2-independent, we tried to identify the potential receptors. We first explored the expression of the known SARS-CoV-2 receptors or co-factors that have been identified in primary T cells from public single-cell NGS data^14^ and in Jurkat T cells in RNA-seq analysis with or without activation, including ACE2/TMPRSS2, AXL, NRP1, KIM-1/TIM-1, ASGR1, and KREMEN1.^18–20^ Moreover, ITGB2 (leukocyte-associated molecule-1, LFA-1), the leukocyte cell Adhesion molecule, has been suggested binding to SARS-CoV-1 ORF7a.^21^ As SARS-CoV-2 shares similar ORF7a as SARS-CoV-1, it would be interesting to evaluate whether LFA-1 also mediated SARS-CoV-2 infection of T cells.

**Fig. 5:**
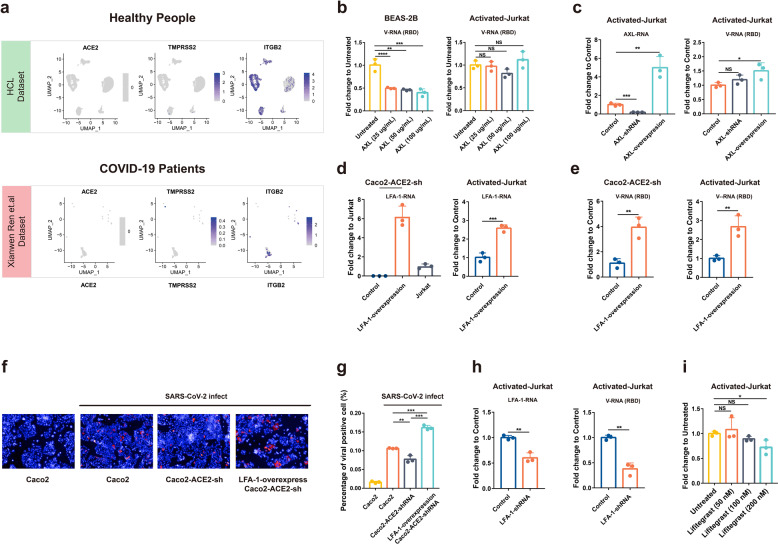
Exploration of potential receptors in T cells. **a** The expression of ACE2, TMPRSS2, and ITGB2 (LFA-1) in blood T cells from healthy donors and COVID-19 patients. The analysis is dependent on public single-cell NGS data.^14^ The expression of the three genes is indicated in SARS-CoV-2 viral RNA-positive T cells for patient penal. **b** BEAS-2B and activated Jurkat cells were incubated with different concentrations of AXL proteins (25, 50, or 100 μg/ml) at 37 °C for 30 min before infected by SARS-CoV-2 (MOI of 0.01). Intracellular viral RNA (RBD) at 24 h post infection was quantified using qPCR. **c** Knockdown or overexpression of AXL were performed on Jurkat cells and the expression level of AXL was detected using qPCR. Cell lines were infected by SARS-CoV-2 at an MOI of 0.01 for 24 h and viral RNA was quantified. **d** LFA-1 was stably overexpressed on ACE2 knockdown Caco2 (Caco2-ACE2-shRNA) or Jurkat cells, and the RNA level was quantified using qPCR. **e** Caco2, activated Jurkat and their respective LFA-1-overexpression cells were infected by SARS-CoV-2 at an MOI of 0.01 and harvested at 24 h post infection. Intracellular viral RNA was detected using qPCR. **f**, **g** Caco2, Caco2-ACE2-shRNA, and its LFA-1 overexpression cell line were infected by SARS-CoV-2 at an MOI = 5 for 8 h. The high content microscope was used to observe (**f**) and quantify (**g**) the viral NP-positive cells. **h** LFA-1 knockdown Jurkat cells were infected with SARS-CoV-2 (MOI = 0.01) for 24 h, and the expression of LFA-1 and intracellular viral RNA was analyzed using qPCR. **i** Activated Jurkat cells were pretreated with Lifitegrast, an inhibitor of LFA-1 at different concentrations (50, 100, or 200 nM) at 37 °C for 30 min before infection (MOI = 0.01). Intracellular viral RNA was quantified at 24 h post infection. The statistics was performed using Student’s *t* test (*P < 0.05; **P < 0.01; ***P < 0.001; ****P < 0.0001; NS no significance)

Our data showed minimal expression of the following molecules in SARS-CoV-2-positive T cells from patients: ACE2, TMPRSS2, ASGR1, KREMEN1, and NRP1 (Fig. 5a and Supplementary Fig. S3a). In contrast, AXL and LFA-1 were expressed in these cells. In Jurkat cells, LFA-1 also showed very high expression, although it was not upregulated following a 2 h activation (Supplementary Fig. S3b). Taken together, AXL and LFA-1 appeared to be promising targets as entry molecules.

AXL was proposed to be a candidate receptor for SARS-CoV-2 in a previous study and the function in mediating SARS-CoV-2 infection is independent of ACE2.^19^ BEAS-2B that was used as a positive control for AXL-SARS-CoV-2 studies was pretreated with AXL proteins of different concentrations (25, 50, 100 μg/ml) for 30 min and then infected with SARS-CoV-2. The infection of SARS-CoV-2 could be significantly inhibited by AXL protein at a concentration of 25 μg/ml. In contrast, SARS-CoV-2 infection of Jurkat cells could not be inhibited even at 100 μg/ml (Fig. 5b). Next, we constructed AXL-knockdown or overexpression cell lines on Jurkat cells and then tested the effect on viral infection. Our data showed that AXL knockdown could not block SARS-CoV-2 infection, but an AXL overexpression could slightly enhance the infection (1.5-fold) (Fig. 5c). Taken together, AXL should not be a main receptor for SARS-CoV-2 in Jurkat cells but it may contribute to infection.

LFA-1 is widely expressed on the surface of many leukocytes, and T-cell activation changed the structure of LFA-1 to a high-affinity mode, but not expression level.^22^ We then overexpressed the high-affinity alpha subunit of LFA-1 protein in ACE2 knockdown Caco2 cells (Caco2-ACE2-shRNA) and Jurkat cells. Our qPCR data showed that the LFA-1 overexpression successfully restored the dampened infection in ACE2 knockdown Caco2 cells, and also enhanced viral infection in Jurkat cells (threefold increase), as shown in cellular viral RNA levels (Fig. 5d, e). To corroborate the finding, we also performed IFA to detect viral NP expression. After an 8 h infection, viral NP-positive cells were compared. Our data showed a dampened SARS-CoV-2 infection in ACE2-knockdown cells, and a much higher NP in LFA-1 overexpression ACE2-knowckdown cells (Fig. 5f, g).

Finally, the LFA-1-knockdown Jurkat cell line was constructed and infected by SARS-CoV-2 (MOI = 0.01). At a 24 h post infection, viral load in the knockdown cell line was significantly decreased compared to the control cell line (Fig. 5h). Lifitegrast, an inhibitor that blocked LFA-1 binding to its extracellular ligand, was also used to pretreat activated Jurkat cells before infection. The qPCR results showed that at a concentration of 200 nM, Lifitegrast could also reduce the viral load in Jurkat cells (Fig. 5i). Collectively, our results suggested that LFA-1 should be an attachment factor or potential entry molecular for SARS-CoV-2 during its infection in Jurkat cells.

## Discussion

Here, we showed that SARS-CoV-2 infected T lymphocytes, mainly CD4 + T cells, in an ACE2-independent manner. SARS-CoV-2 infection triggered pronounced T-cell death, which potentially contributed to lymphopenia in patients with COVID-19. T-cell infection may also pose profound influences on patients. Infected T lymphocytes not only lost the ability to control viral infection but may also carry viruses to other parts of the body through blood circulation. In addition, this ACE2-independent infection mode may compromise the therapeutic effect of neutralizing antibodies targeting at spike-ACE2 binding. These may synergistically result in more severe infection outcomes in patients with COVID-19.

It has been debated whether SARS-CoV-2 impaired the functionality of immune cell populations through direct infection. Our results provided evidence to show that SARS-CoV-2-infected T cells, as viral RNA, viral sgRNA, viral protein, and the infectious virus could be detected from T cell upon infection or from patient PBCs, although the production of infectious virus particles may stay at a low level. Several recent studies also revealed that multiple immune cells carry viral antigen or viral RNA, including neutrophils, macrophages, inflammatory monocytes, plasma B cells, T cells, and NK cells through postmortem histology analysis and single-cell/single-nuclear RNA-seq to lung or BALF.^13–15^ This suggests that SARS-CoV-2 should have a broad tropism of target cells, including major immune cells populations.

Human ACE2 and TMPRSS2 proteins were recognized as the main proteins that mediated SARS-CoV-2 cell entry.^14^ The newly discovered binding molecules AXL and NRP1 are still dependent on ACE2 as the main receptor.^18,19^ Our discovery of ACE2-independent infection of T cells is surprising, but is also supported by previous discoveries that there are SARS-CoV-2 RNA^+^ cells which did not co-express ACE2 and TMPRSS2.^15^ In our data, SARS-CoV-2 showed significant infection of activated T cells, suggesting there should be a new entry mechanism in T cells. The identification of LFA-1, as an entry molecule that contributed to a SARS-CoV-2 infection of T cells would be important for developing clinical therapeutics, although future questions remain. For example, what is the LFA-1 binding protein in SARS-CoV-2 virion if it is not the spike protein. Since LFA-1 is expressed in a number of other leukocytes, it can be expected that other immune cells (including macrophages or monocytes) could also be infected by SARS-CoV-2 potentially through binding with LFA-1. These questions should be addressed in future studies.

The infection of CD4 + T lymphocytes by SARS-CoV-2 virus may be a major contributor of virus induced pathogenesis. Armed T cells play a pivotal role against pathogen infection.^10^ As shown in our data, these T cells are likely to be targets of SARS-CoV-2 infection and undergo apoptosis in the HIF-1a-dependent pathway. These events may lead to T-cell dysfunction, depletion, and eventually lymphopenia in patients. In addition, the dying CD4 + T lymphocytes could trigger excessive inflammation that leads to severe immunopathogenesis in patients. Notably, the population of CD8 + T lymphocytes is also significantly decreased in COVID-19 patients. Unlike CD4 + T lymphocytes, these cells were not determined to contain SARS-CoV-2 viral antigen in flow cytometry. The mechanism underlying SARS-CoV-2 infection-induced CD8 + T lymphocytes depletion is currently unknown. Besides viral infection, several mechanisms, including the presence of endogenous or exogenous glucocorticoids, over-activated neutrophil releasing inhibitors of T cell activation (Arginase 1 and CD274) and cytokine-regulated selective differentiation of bone marrow cells, might also contribute to lymphocytes depletion.^11,23^ Further in-depth investigation is needed to address the potentially multi-mode mechanisms that lead to lymphopenia in the COVID-19 patients. Considering the apparent correlation between lymphopenia and disease progression in COVID-19 patients, it is important to develop strategies to prevent virus-induced lymphopenia.

## Materials and methods

### Samples and ethics

Human blood and tissue samples from patients with COVID-19 or from healthy donors were collected by Tongji hospital with consent from all persons. Fresh lung biopsy sections were prepared from a deceased patient. The ethics committee of the designated hospitals for emerging infectious diseases approved all human samplings.

### Cell lines and virus culture

Vero E6, Caco2, 293T-sg, GP2-293, and BEAS-2B in DMEM + 10% FBS, or MT4 and Jurkat T cells in RPMI1640 + 10% FBS (Gibco, C22400500BT), or A549 cells in DMEM/F12 + 10% FBS, or primary T cells in X-vivo (Lonza, 04-418Q) medium containing IL-2 (Peprotech, 200-02) were cultured at 37 °C in a humidified atmosphere of 5% CO_2_. All cell lines were tested free of mycoplasma contamination and applied to species identification and authenticated by microscopic morphologic evaluation. None of cell lines was on the list of commonly misidentified cell lines (by ICLAC). SARS-CoV-2 isolate WIV04 (GISAID accession number EPI_ISL_402124) was used in this study. WIV04 was isolated from Huh7 cells from the original sample and was passaged in Caco2 cells. Viral titer (TCID50/ml) was determined in Vero E6 cells.

### Proteins and antibodies for SARS-CoV-2

SARS-CoV-2 strain WIV04 NP and predicted RBD were inserted into pCAGGS vector with an N-terminal S-tag. Constructed plasmids were transiently transfected into HEK293T-17. Supernatant collected for protein purification was purified using S-tag resin, the purity and yield were tested using anti-S-tag mAb (generated in-house). Rabbits were immunized with purified NP proteins three times at a dose of 700 ng/each, 2 weeks interval. Rabbit serum was collected at 10 days after the final immunization. Antibody titer was determined in an ELISA using purified NP protein as a detection antigen.

### Peripheral blood cells (PBCs) preparation and SARS-CoV-2 infection

The blood samples from patients with COVID-19 or healthy donors were processed in BSL3 lab at WIV. In all, 1× RBC lysis buffer was made from eBioscience™ 10× RBC Lysis Buffer before the experiment (Multi-species, Invitrogen). Human blood samples were centrifuged at 500 × *g* for 10 min before being treated with 2 ml 1× RBC lysis buffer for no more than 15 min at room temperature. Cells were spun down at 500 × *g* for 10 min, followed by treatment using 2 ml 1× RBC lysis buffer for another 10 min at room temperature to remove the residue red blood cells. Cells were ready for use after centrifugation. Cells were spin washed (500 × *g* for 10 min each time) three times with PBS containing 2% BSA before staining of cell marker antibodies.

For infection, PBCs were seeded into 24-well plates in Roswell Park Memorial Institute 1640 culture medium (RPMI1640, ThermoFisher, 22400500BT) supplemented with 10% fetal bovine serum (FBS, Life Technologies, 10099141) at a density of 1 × 10^6^ cells/ml. PBCs were infected with SARS-CoV-2 at 0.1 MOI. One hour after incubation, cells were spin washed for three times using RPMI1640. PBCs were then seeded with RPMI1640 supplemented with 10% FBS in new 24-well plates at 37 °C supplied with 5% CO_2_ for 12 h or 24 h before being collected for further analysis.

For IFA on patient PBCs, overnight fixed cells were evenly smeared over a glass coverslip. The presence of viral NP was detected with rabbit pAb against the SARS-CoV-2 NP protein (generated in-house, 1:1000) and a Cy3-conjugated goat anti-rabbit IgG (1:200, Abcam, ab6939). T lymphocytes were detected using a rabbit anti-human CD3 antibody (1:100, Abcam, ab5690). Nuclei were stained with DAPI (Beyotime, C1002). Staining patterns were examined using confocal microscopy on a FV1200 microscope (Olympus).

For immunohistochemistry analysis on patient lung, the biopsy tissues from a deceased patient were fixed with 4% paraformaldehyde for 24 h, paraffin-embedded and cut into 5-μm sections. Multiplex immunofluorescence staining was obtained using PANO 7-plex IHC kit (0004100100, Panovue, Beijing, China). Slides were deparaffinized and rehydrated, followed by 15-min heat-induced antigen retrieval with EDTA pH 9.0. The slides were washed with PBS/0.02% Triton X-100 then blocked with 10% BSA at RT for 30 min, rabbit pAb against the SARS-CoV-2 NP protein (generated in-house, 1:1000) and rabbit anti-human CD3 antibody (1:100, Abcam, ab5690) were then used in incubation at 37 °C 1 h, followed by horseradish peroxidase-conjugated secondary antibody incubation and tyramide signal amplification. The slides were microwave heat-treated after each TSA operation. Nuclei were stained with 4’-6’-diamidino-2-phenylindole (DAPI, Beyotime, C1002) at the final stage of staining. To obtain multispectral images, the stained slides were scanned using the Mantra System (PerkinElmer). The scans were combined to build a single stack image. Unstained images and single-stained sections were used to extract the spectrum of autofluorescence of tissues or each fluorescein, respectively. The extracted images were further used to establish a spectral library required for multispectral immixing by InForm image analysis software (PerkinElmer). Using this spectral library, we obtained reconstructed images of sections with the autofluorescence removed.

### PBCs co-cultured with Caco2 cells

PBCs from five patients were washed three times with PBS before co-cultured with Caco2 cells for 4 days and tested for SARS-CoV-2 viral RNA in the supernatant or antigen in Caco2 cells. In the meantime, PBCs were analyzed for the presence of viral antigen by flow cytometry.

### Human colon organoids culture and SARS-CoV-2 infection

Human colon organoids were generated and cultured as described in the previous study.^24^ Briefly, colon organoids in matrigel were digested and washed twice with medium before infection. SARS-CoV-2 was added to infect colon organoids at an MOI of 0.01. 24 h later, colon organoids were then spun down and washed twice with medium. Viral RNA in colon organoids was determined by qPCR.

### Activation of Jurkat and primary T cells

To activate Jurkat cells, 2.5E + 06 of cells were seeded to a well of a six-well plate containing 2.5 ml of RPMI1640 medium containing 10% FBS. In total, 40 ng/ml of PMA (Invivogen, tlrl-pma) was added to cells and incubated at 37 °C for 2 h. Cells were centrifuged at 300 × *g* at room temperature for 10 min before discarding the supernatant and cultured with fresh RPMI1640 medium containing 10% FBS. Primary human CD3 T lymphocytes were isolated from blood of healthy donors using CD3 Microbeads of Human (Miltenyi, 130-050-101). To activate primary T cells, frozen T cells were thawed and cultured with X-vivo (Lonza, 04-418Q) containing 1 μg/ml of IL-2 (Peprotech, 200-02). Cells were cultured with a volume of 7.5 μl of T Cell TransAct (Miltenyi Biotec) in the medium for 3 days at 37 °C. Cells were then spun down and cultured with fresh IL-2/X-vivo medium before viral infection.

### T-cells infection

Jurkat T cells or primary human CD3 T lymphocytes were infected with SARS-CoV-2 at a MOI of 0.01, 0.1, or 1 depending on the purpose of the experiment. Supernatant or cells were harvested at 0, 24, 48, or 72 hpi after three times PBS washing for Jurkat T cells, or 0, 4, 8, and 12 hpi for primary T cells. Cellular or supernatant viral RNA or protein expression was determined by qPCR, RNA-seq, WB, or flow cytometry. GAPDH was used in qPCR as internal control and beta-tubulin was used in WB (1:5000, 66240-1-Ig from Proteintech) as an internal control.

### Flow cytometry analysis of human peripheral blood samples

For surface staining, PBCs were incubated with fluorochrome-labeled antibodies specific for humans before fixation: AF-700-anti-CD45 (2D1), percp-anti-CD19 (HIB19), APC/CY7-anti-CD3 (UCHT1), BV510-anti-CD4 (OKT4) and percp/Cy5.5-anti-CD8a (HTT8a). Antibody-stained PBCs were fixed overnight with 4% PFA at 4 °C and taken out of BSL3 lab for downstream analysis. Cells were stained further with in-house-made SARS-CoV-1 NP pAb (1:500) at 4 °C for 30 min after permeabilization. Then cells were stained with FITC-anti-Rabbit IgG (H + L) at room temperature for 30 min. AF-700-anti-CD45 (2D1), APC/CY7-anti-CD3 (UCHT1), BV510-anti-CD4, and percp/Cy5.5-anti-CD8a antibodies were purchased from Biolegend and all were used at 1:100. FITC-anti-Rabbit IgG (H + L) was from Proteintech (SA00003-2).

### RNA extraction and qPCR

Whenever commercial kits were used, the manufacturer’s instructions were followed without modification. RNA was extracted from 140 μl of samples with the QIAamp^®^ Viral RNA Mini Kit (QIAGEN). RNA was eluted in 50 μl of elution buffer and used as the template. The qPCR detection of SARS-CoV-2 was performed using HiScript^®^ II One-Step qPCR SYBR^®^ Green Kit plus One-Step qPCR Probe kit targeting at either M for sgRNA (designed in house) or RBD of spike gene (commercial) following the instructions of the manufacturer (Q222-CN, Vazyme Biotech Co., Ltd). QPCR was run in a Step-One Plus real-time PCR machine (ABI) machine using default settings.

### SARS-CoV-2 genome depth and coverage analysis

RNA was extracted from SARS-CoV-2 24 h-infected activated Jurkat T cells with the RNAprep Pure Cell/Bacteria Kit (TIANGEN, DP430). RNA was eluted in 50 μl of elution buffer and used as the template for RNA-seq. Clean reads were mapping to SARS-CoV-2 genome (WIV04) using software HISAT2 v2.1.0. After sorted and indexed with samtools v1.10-24, the coverage was calculated using genomeCoverageBed function from bedtools v2.29.2.

### Transcriptome analysis

The SARS-CoV-2 24 h- and 48 h-infected Jurkat T cells (3 replicates each), blood samples from three healthy donors, and 3 severe COVID-19 patients were subjected for RNA-seq analysis. After mapping clean reads to GRCh38.p13 with HISAT2 v2.1.0 and format conversion with samtools v1.10-24, we used stringtie v2.1.0 to assemble and quantitate transcripts. Reads counts table of transcriptome generated by prepDE.py, a tool in stringtie, was used for gene differential expression analysis in R v4.1.0 with package DESeq2 v1.32.0. The gene with log2 fold change >2 and *P* value <0.05 was selected to perform enrichment analysis using online tools Metascape.

### Public single-cell NGS data analysis

Public single-cell NGS data were downloaded, COVID-19 patients’ data were downloaded from GSE158055^14^ and healthy donors’ data were from GSE134355 (human cell landscape). According to the original information of each article, we extracted data of primary T cells from lung, thymus, and peripheral blood of healthy donors and virus-positive T cells of COVID-19 patients. Following the standard Seurat v4.0.4 workflow, we normalized the data and scaled it with UMI information. The expression of candidate receptors or co-factors was visualized with Seurat function FeaturePlot.

### Western blot (WB) analysis

Infected or transduced cells were harvested at the indicated time point and lysed with RIPA Lysis Buffer (Beyotime, P0013C) for WB. Proteins in cell lysates were then separated on 10–12% SDS-polyacrylamide gel electrophoresis (PAGE) and further transferred to polyvinylidene difluoride (PVDF) membranes (Millipore, SLHVR33RB). Blots were incubated with rabbit polyclonal anti-ACE2 (Servicebio, GB11267, 1:1000 dilution), rabbit polyclonal anti-2019-nCoV NP (1:1000 dilution), mouse monoclonal anti-beta-tubulin (Proteintech, 66240-1-Ig, 1:5000 dilution), and then appropriate rabbit or mouse peroxidase-conjugated secondary antibodies (Proteintech, 1:5000 dilution, SA00001-2, or SA00001-1). Immobilon western chemiluminescent HRP substrate (Millipore, WBKLS0500) was used for protein detection.

### Terminal deoxynucleotidyl transferase dUTP nick end labeling (TUNEL) assay

The TUNEL Assay kit purchased from Beyotime Biotechnology (C1088) was used to detect apoptosis in SARS-CoV-2-infected cells according to the manufacturer’s instructions. Briefly, cells fixed in 4% paraformaldehyde were permeabilized with 0.25% Triton X-100 for 20 min at 4 °C. Then the TdT reaction mixture containing TdT enzyme and fluorescent labeling solution was added to the cells to label the fragmented DNA. Cells were further stained with Rp3-CoV NP pAb (1:8000) or Rabbit anti-SARS-CoV-2 NP pAb (1:500) and CY3-anti-Rabbit IgG (H + L) (Proteintech, SA00009-2) after fixation. Labeled cells were analyzed with a flow cytometer (BD LSRFortessa).

### ACE2 competition inhibition and antibody blocking experiments

Human recombinant full-length ACE2-Fc protein (GenScript, Z03484), Anti-ACE2 Ab (R&D, AF933) and RD#4-anti-Spike Ab (house-made monoclonal antibodies) were used. ACE2-Fc protein was diluted to 20 μg/μl in culture medium and then incubated with SARS-CoV-2 virus solution (MOI = 0.01) at a volume of 1:1 at 37 °C for 30 min. The RD#4-anti-Spike Ab was diluted to 320 ng/μl in culture medium and then incubated with SARS-CoV-2 virus solution (MOI = 0.01) at a volume of 1:1 at 37 °C for 30 min. The virus-ACE2 or virus–antibody mixtures were then added to Jurkat cells or Caco2 cells. Cells were collected for further analysis at 24 h post infection. For anti-ACE2 antibody blocking experiments, Jurkat cells or Caco2 cells were pretreated with 3.33 ng/μl anti-ACE2 antibody (R&D Systems, goat, AF933) at 37 °C for 30 min before infection.

### Generation of KO, KD, overexpression cell lines

KO, KD, and overexpression plasmids were constructed on different vectors (pLenti-V2 for knockout, pLKO.1 vector for knockdown, and pQCXIH vector for overexpression). Knockout of ACE2 was accomplished by transduction of Caco2 and Jurkat cells with lentiviruses expressing specific sgRNAs targeting ACE2 (F: CACCG GCCTCCATCGATATTAGCAA; R: AAAC TTGCTAATATCGATGGAGGCC).

Knockdown of ACE2, AXL, LFA-1 was accomplished by transduction of Caco2 or Jurkat cells with lentiviruses expressing specific siRNAs (ACE2: 5′-GCCGAAGACCTGTTCTATCAA-3′; AXL: 5′- CCTGTGGTCATCTTACCTT-3′; LFA-1: 5′-GCCATCAATTATGTCGCGACA-3′ or scramble siRNA).

Then the transduced cells were cultured with puromycin (5 μg/ml for Caco2 or 1.5 μg/ml for Jurkat) for 7 days.

For overexpression, the full length of AXL or domain I of LFA-1 alpha subunit were amplified from Hep G2 cells or Jurkat cells respectively. Lentivirus transduced cells were cultured with hygromycin (35 μg/ml for Caco2 and Jurkat cells) for 7 days. For the infection, virus was added to the cells until the end of the experiment with 0.01 MOI. Infected cells were harvested at 24 hpi after twice washing with PBS. Intracellular viral protein expression was determined by western blotting assay with antibody against virus NP protein and viral RNA in the cytoplasm was determined by qPCR.

### TMPRSS2 blocking assay

Camostat mesylate (MCE, HY-13512-10 mM) was diluted to a final concentration of 20 μM or 2 μM. In total, 100 μl (for a 48-well plate) or 200 μl (for a 24-well plate) of Camostat solutions were added to cells. One hour later, activated Jurkat and Caco2 cells were infected with SARS-CoV-2 at 0.01 MOI. The cell lysate was harvested at 24 hpi and viral RNA in the cytoplasm was determined by qPCR. Viral NP was analyzed by western blot.

### Candidate receptor proteins competition inhibition experiments

Recombinant Human AXL Protein (MedChemExpress, HY-P7622) was diluted to different concentrations with culture medium and then incubated with SARS-CoV-2 virus (MOI = 0.01) at a volume of 1:1 at 37 °C for 30 min. Mixtures were then added to infect activated Jurkat cells and BEAS-2B cells. Samples were harvested at 24 hpi and cellular viral RNA was determined by qPCR.

### LFA-1 inhibition experiment

Lifitegrast (MedChemExpress, HY-19344) was diluted to different concentrations and pretreated activated Jurkat cells at 37 °C before infection. Thirty minutes later, cells were infected with SARS-CoV-2 (MOI = 0.01) and samples were harvested at 24 hpi. Viral RNA in the cytoplasm was determined by qPCR.

### Electron microscopy

Activated Jurkat and MT4 cells were infected with the SARS-CoV-2 (MOI = 1) for 72 h. Cells were collected and fixed with 2.5% (w/v) glutaraldehyde and 1% osmium tetroxide, dehydrated through a graded series of ethanol concentrations (from 30 to 100%), and embedded with epoxy resin. Ultrathin sections (80 nm) of embedded cells were prepared, deposited onto Formvar-coated copper grids (200 mesh), stained with uranyl acetate and lead citrate, and analyzed using a 200-kV Tecnai G2 electron microscope.

### Statistical analysis

Data analyses were performed using GraphPad Prism 7.0 software. Data were shown as mean ± SD. Data were analyzed with Shapiro–Wilk normality test and confirmed to the Gaussian distribution. Statistical analysis was performed using Student’s *t* test with two-tailed, 95% confidence. *P* values less than 0.05 were considered statistically significant.

## Supplementary information

Supplementary Materials for ACE2-independent infection of T lymphocytes by SARS-CoV-2

## Data Availability

Data presented in this study are available on request from the corresponding authors. The data are not publicly available due to limitations in the material transfer agreement.
