# Supplementary material for: ACE2-independent infection of T lymphocytes by SARS-CoV-2
Source: Signal Transduct Target Ther. 2022 Mar 11;7:83. doi: 10.1038/s41392-022-00919-x (PMC8914143; doi:10.1038/s41392-022-00919-x)
Supplement: Supplementary file 1 — Supplementary Materials for ACE2-independent infection of T lymphocytes by SARS-CoV-2 [file 41392_2022_919_MOESM1_ESM.docx]

Supplementary Materials for

ACE2-independent infection of T lymphocytes by SARS-CoV-2

Xu-Rui Shen, Rong Geng, Qian Li , Ying Chen, Shu-Fen Li, Qi Wang, Juan Min, Yong Yang, Bei Li, Ren-Di Jiang , Xi Wang, Xiao-Shuang Zheng, Yan Zhu, Jing-Kun Jia, Xing-Lou Yang, Mei-Qin Liu, Qian-Chun Gong, Yu-Lan Zhang, Zhen-Qiong Guan, Hui-Ling Li, Zhen-Hua Zheng, Zheng-Li Shi, Hui-Lan Zhang, Ke Peng and Peng Zhou

Correspondence to: [peng.zhou@wh.iov.cn](mailto:peng.zhou@wh.iov.cn); [pengke@wh.iov.cn](mailto:pengke@wh.iov.cn); [huilanz_76@163.com](mailto:huilanz_76@163.com).

**This PDF file includes:**

Figures. S1 to S3

Figure. S1


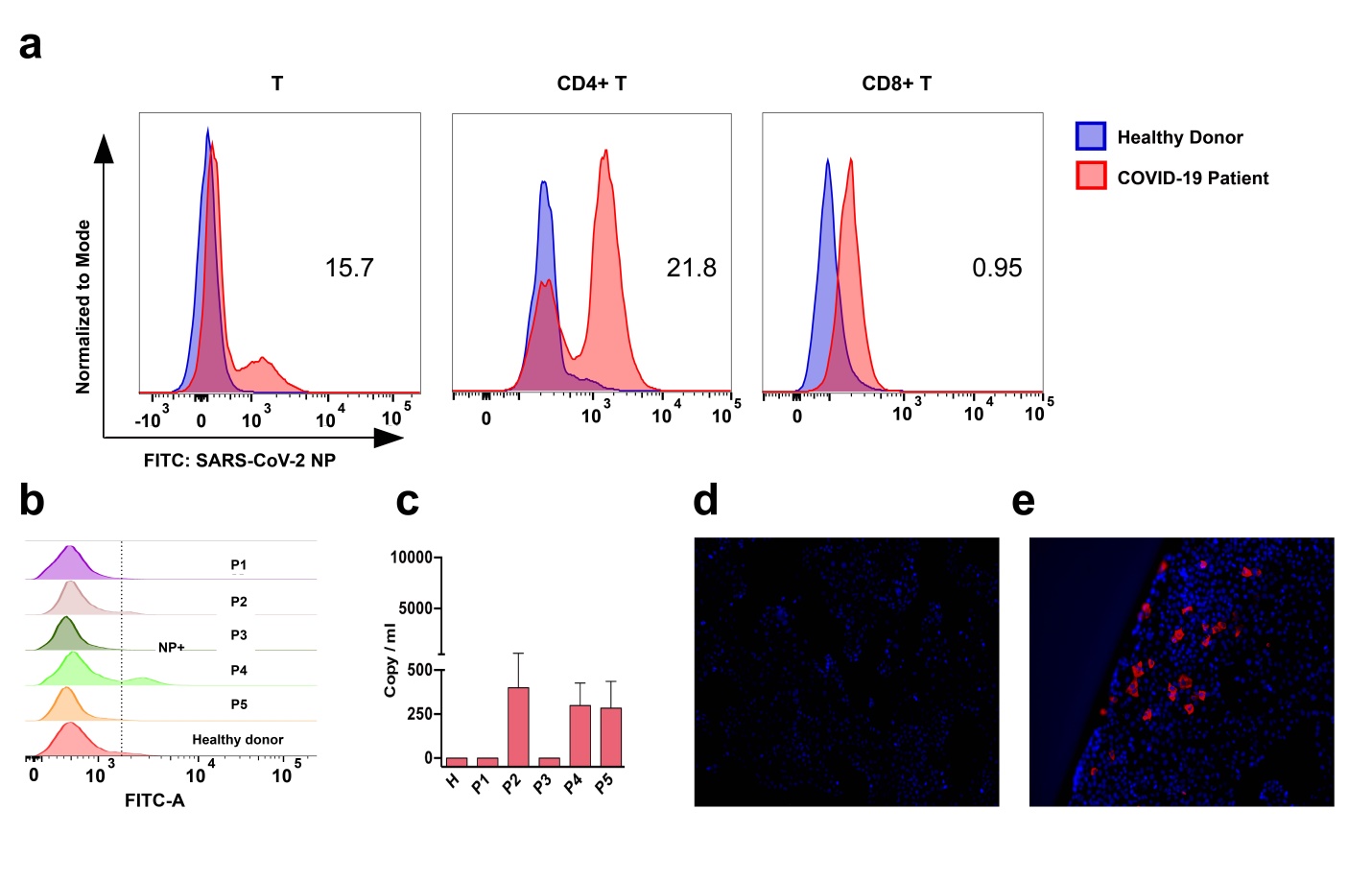


**Fig. S1**. **PBCs from patients with COVID-19 carry infectious SARS-CoV-2 virus.** (**a**) Presence of SARS-CoV-2 NP protein in T lymphocytes. Blue, cells from a healthy donor; red, cells prepared from a COVID-19 patient. Percentage of viral positive cells was shown. (**b-e**) Co-culture of viral positive PBCs with Caco2 cells. PBCs from five COVID-19 patients (P1-P5) or from one healthy donor (H) were washed three times before used in flow cytometry analysis of viral infection (**b**), viral positivity was shown for P2 and P4) or co-cultured experiment. After four days co-culturing, viral RNA in cell culture supernatant was tested in qPCR (**c**), and viral NP protein was detected from healthy donor (**d**) or P4 sample (**e**) co-cultured Caco2 cells in IFA. Cells were stained with in-house made SARS-CoV-2 NP pAb (red) and DAPI (blue).

Figure. S2


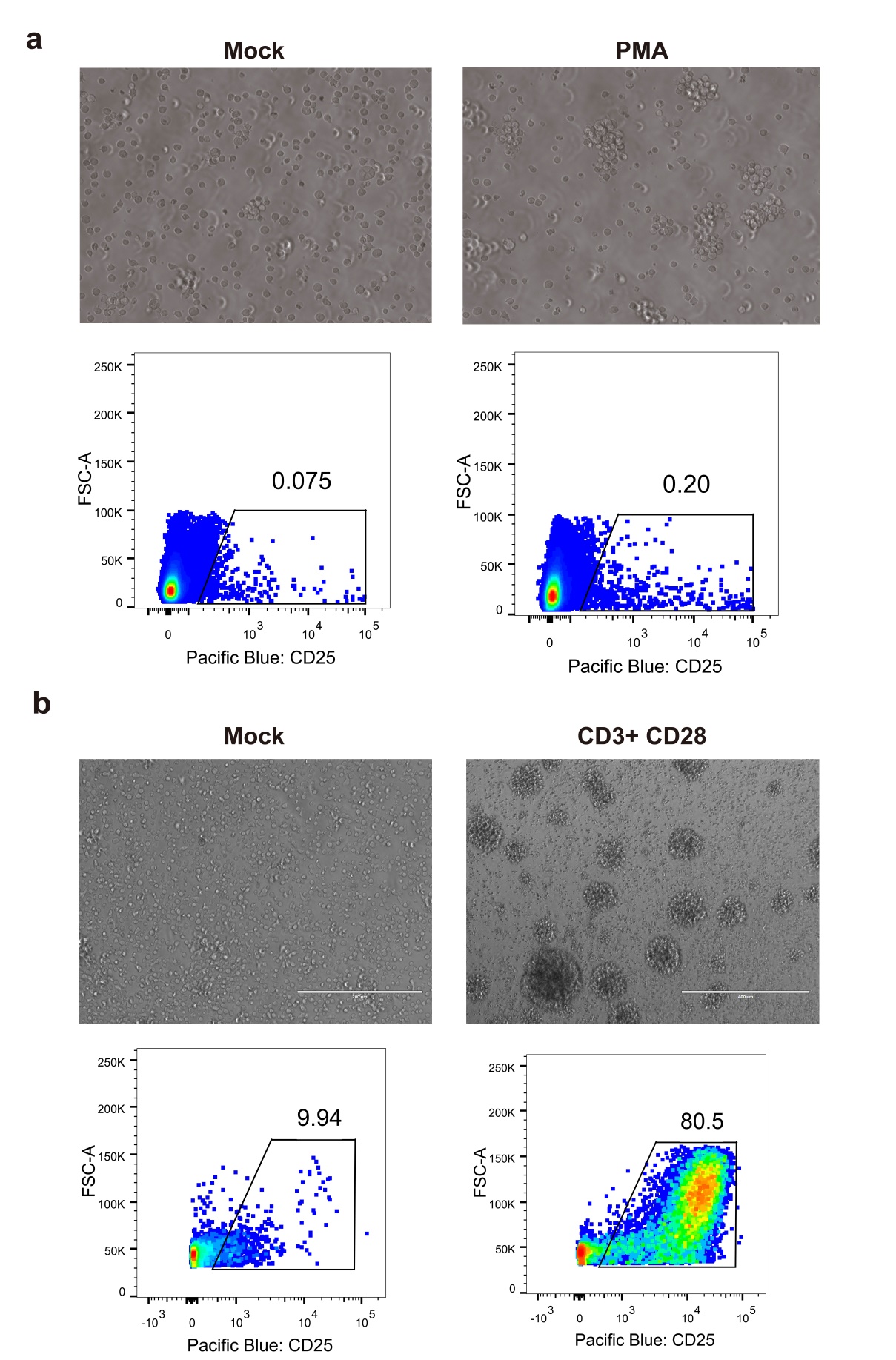


**Fig. S2. Activation of T cells.** (**a**) Jurkat cells were activated by PMA for 2h and the bright-field photos of mock or PMA-treat cells were taken. Expression level of CD25 was determined by flow cytometry. (**b**) Human primary T cells were activated by CD3 and CD28 for 3 days and the bright-field photos of mock or CD3&CD28-treat cells were taken. Expression level of CD25 was determined by flow cytometry.

Figure. S3


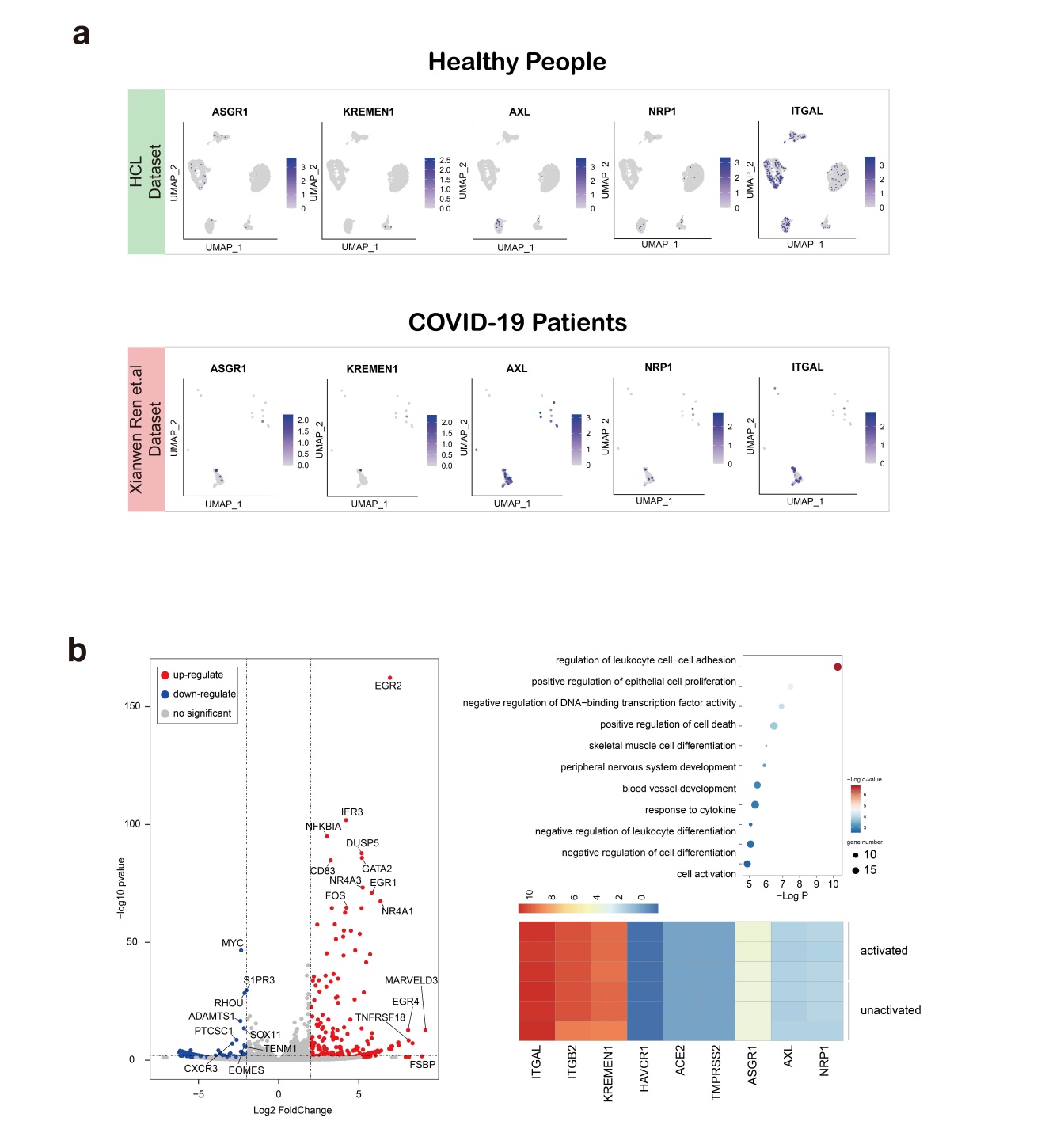


**Fig. S3. Expression of receptor candidates in T cells.**(**a**) Expression of candidate SARS-Cov2 receptors in T cell of healthy donors (green panel) and COVID-19 patients (red panel). (**b**) Unactivated or 2h activated Jurkat cells were subjected to RNA-seq analysis. Volcano plot showed differential expressed genes in activated Jurkat cells compared to unactivated Jurkat cells. Up-regulated pathways were enriched and the expression of candidate SARS-CoV2 receptors were shown in heat map.
